# Supplementary material for: High throughput error corrected Nanopore single cell transcriptome sequencing
Source: Nat Commun. 2020 Aug 12;11:4025. doi: 10.1038/s41467-020-17800-6 (PMC7423900; doi:10.1038/s41467-020-17800-6)
Supplement: Supplementary file 1 — Supplementary Information [file 41467_2020_17800_MOESM1_ESM.pdf]

Supplementary Information

Supplementary figures

Supplementary Figure 1

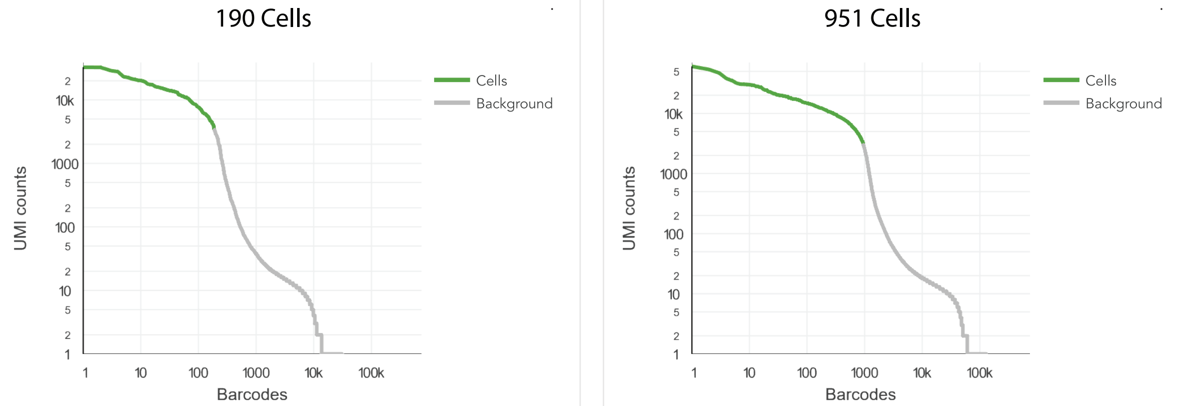

190 Cells	
Estimated Number of Cells	190
Fraction of Reads in Cells	83.8%
Mean Reads per Cell	236,737
Mean Genes per Cell	2,807
Total Genes Detected	14,306
Median UMI Counts per Cell	7,974
Reads Mapped Confidently to Transcriptome	53.4%
Reads Mapped Confidently to Exonic Regions	55.5%
Reads Mapped Confidently to Intronic Regions	17.3%
Reads Mapped Confidently to Intergenic Regions	3.4%
Sequencing Saturation	90.5%

951 Cells	
Estimated Number of Cells	951
Fraction of Reads in Cells	85.1%
Mean Reads per Cell	74,451
Mean Genes per Cell	2,616
Total Genes Detected	16,098
Median UMI Counts per Cell	7,549
Reads Mapped Confidently to Transcriptome	57.0%
Reads Mapped Confidently to Exonic Regions	59.7%
Reads Mapped Confidently to Intronic Regions	17.9%
Reads Mapped Confidently to Intergenic Regions	3.6%
Sequencing Saturation	74.8%

Supplementary Figure 1. Illumina short-read sequencing statistics.

Statistics for the Illumina short read data generated by the 10x Genomics Cell Ranger software are shown for the 190 and the 951 cell replicates.

Supplementary Figure 2

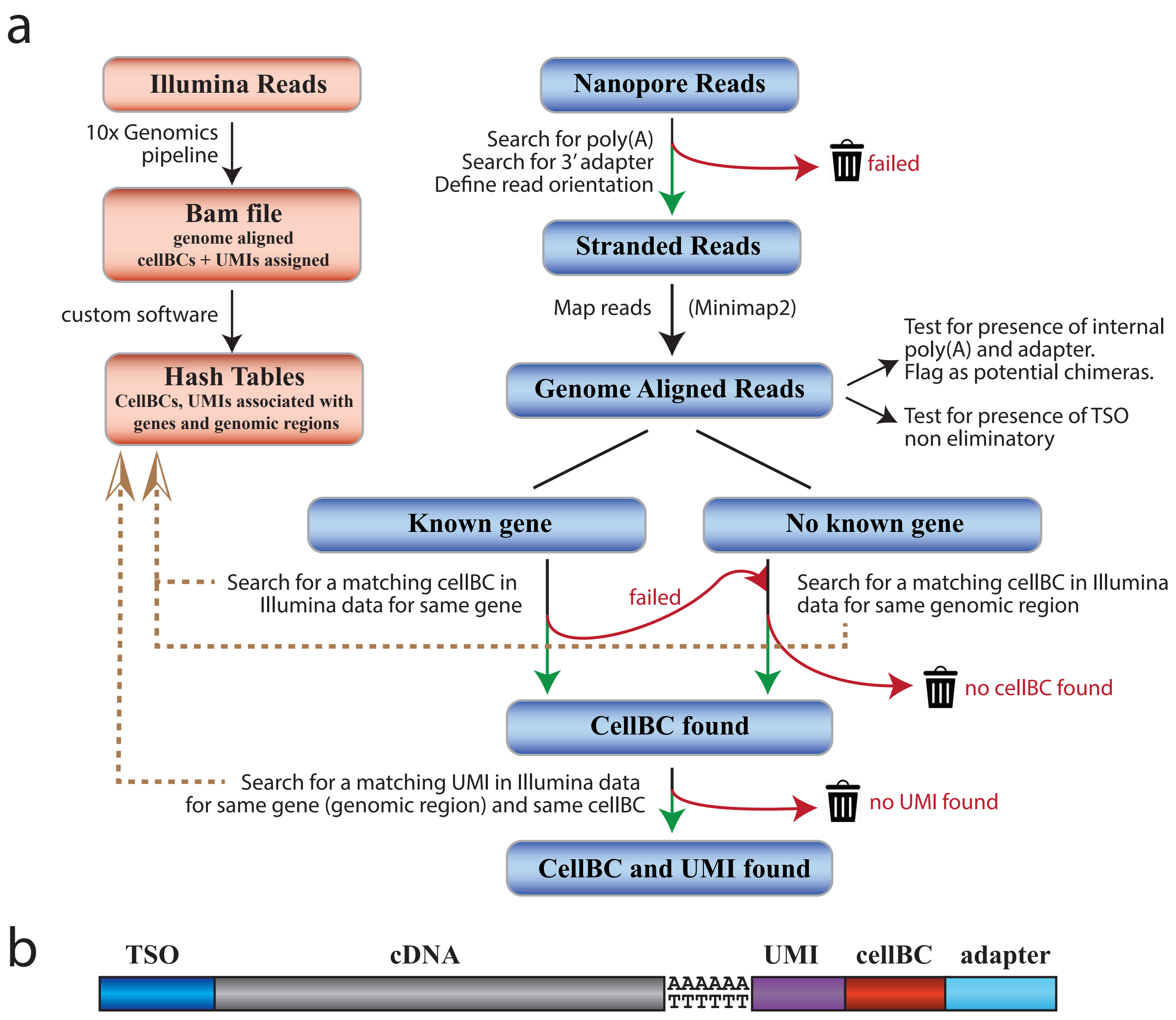

Supplementary Figure 2. CellBC and UMI assignment strategy.

(a) CellBC and UMI assignment strategy. See methods section for description of the individual steps. **(b)** Organization of single cell full-length cDNA generated by the 10x Genomics workflow.

Supplementary Figure 3

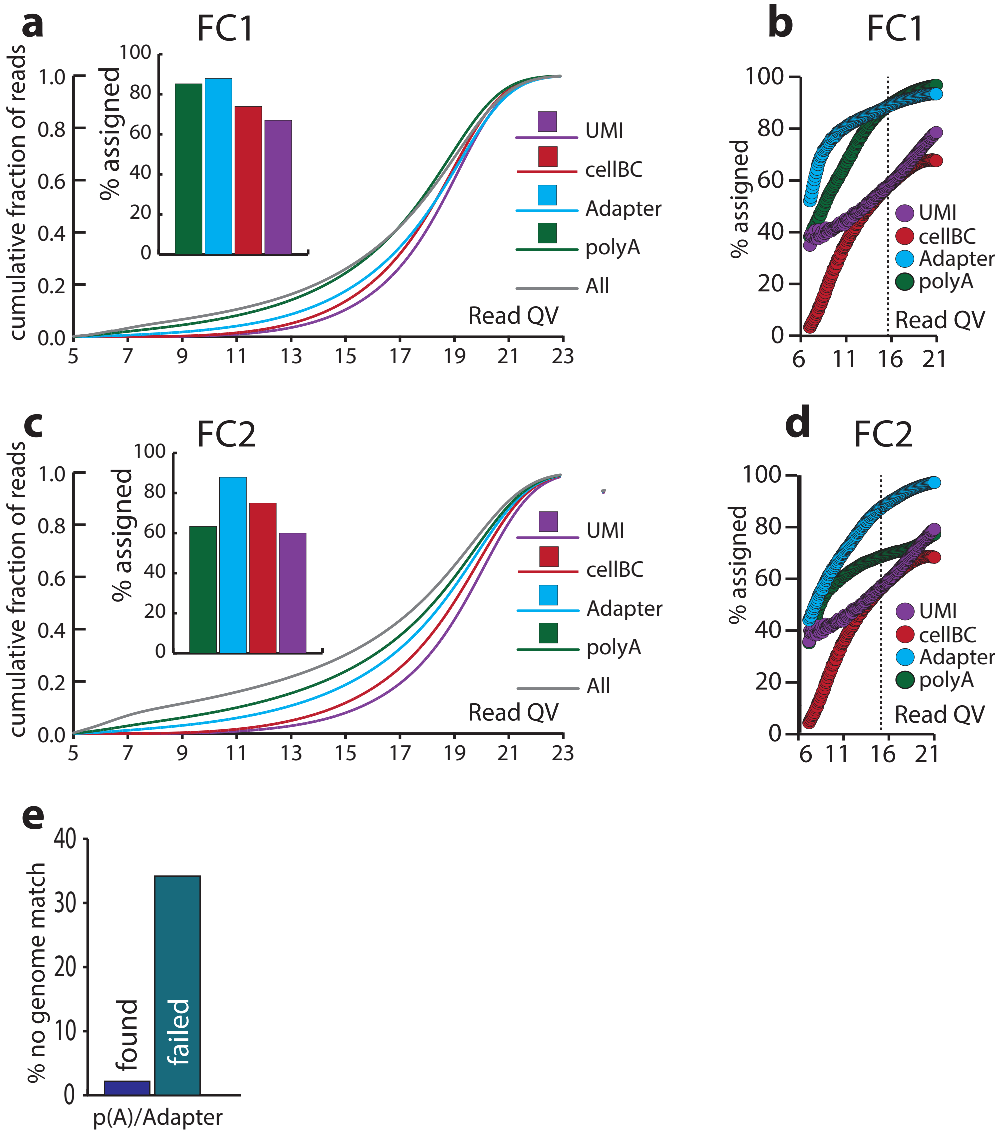

Supplementary Figure 3. Efficiency of cellBC and UMI assignment.

(a,c) Cumulative read quality distributions for two representative Promethion sequencing runs (a: FC1, 32 $*10^6$ reads, 190 cells; c: FC2 70 $*10^6$ reads, 951 cells) are shown for all reads (grey), polyA- (green), adapter- (light blue), cellBC- (red) and UMI- (purple) assigned reads. Fractions of reads (%) in windows of 0.1 (PHRED score) are shown. Histograms show fractions of reads that passed the poly(A) scan, adapter scan, cellBC assignment, UMI assignment. **(b,d**) Relation between read quality and poly(A), adapter, cellBC and UMI assignment efficiency. Dashed vertical lines indicate mean QV of all reads. Poly(A), adapter, cellBC and UMI assignment efficiencies in (a-d) are relative to total reads, adapter assigned reads and cellBC assigned reads respectively. **(e)** The poly(A) scan eliminates the majority of reads that don't match the genome. Fraction of unmapped reads with found poly(A) and adapter (2.2% of 24,008,457) and for reads without identified poly(A) and adapter (35.1% of 8,062,431) for FC1.

Supplementary Figure 4

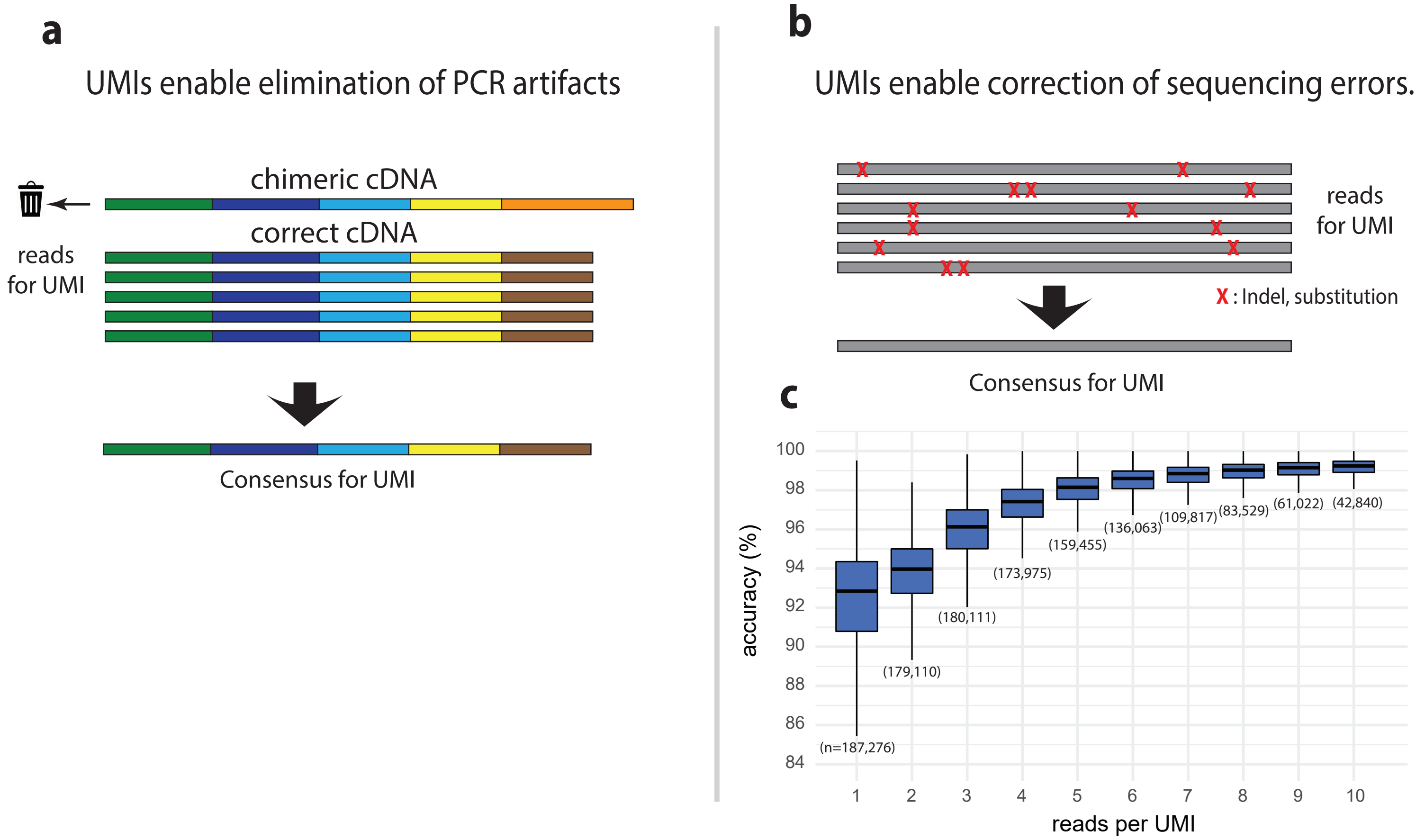

Supplementary Figure 4. UMIs enable identification of PCR artefacts and sequencing error correction.

(a) UMIs allow identification and elimination of reads originating from chimeric cDNA generated during PCR amplification. Chimeric cDNAs are mainly generated in later PCR cycles when cDNA concentration becomes higher. This results in a small fraction of reads ($fraction = \frac{1}{2^n}$, n : PCR cycle where chimera is generated) with aberrant exon layout. Those inconsistent reads are discarded during consensus cDNA sequence definition for the UMI. **(b, c)** UMIs enable sequencing error correction. Generation of consensus sequences for all reads associated with each UMI allows to obtain high accuracy sequences for each UMI (RNA molecule) despite the lower accuracy of individual Nanopore reads. **(c)** the box plots indicate the accuracy of the cDNA consensus sequences (% identity to the reference genome) for different UMI sequencing depths. Boxes represent the 25% quantile to 75% quantile range, upper and lower edges of notches are median +/- 1.58 * IQR / sqrt(n) (IQR: inter quantile range, n: number of UMIs is indicated for each sequencing depth).

Supplementary Figure 5

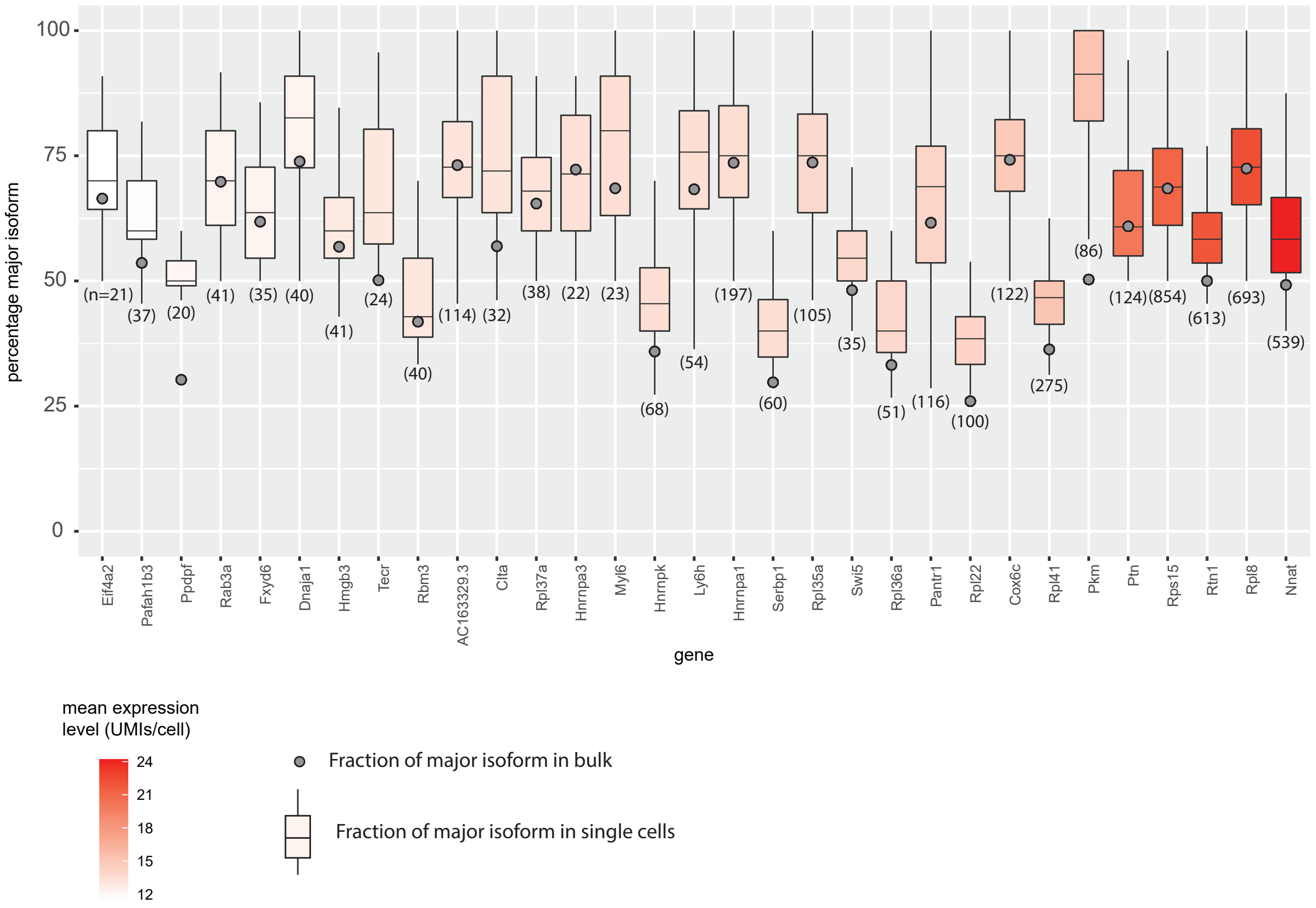

Supplementary Figure 5. Fraction of the most expressed isoforms in single cells.

Boxplots show the fraction of the major isoform in single cells for a selection of genes that fulfill the following criteria: (i) at least 1,000 UMIs for the gene are found in the 1,121 cell dataset. (ii) the major isoform accounted for no more than 75% of the UMIs, to favor genes with high isoform diversity; (iii) genes with a minimum of 10 UMIs present in at least 20 single cells, in order to focus on genes with a sufficiently high expression and low drop-out rates. This signal was compared to the bulk signal (grey dots), which was assessed in the entire dataset. Boxes represent the 25% quantile to 75% quantile range, upper and lower edges of notches are median +/- 1.58 * IQR / sqrt(n) (IQR: inter quantile range, n: sample size).

Supplementary Figure 6

a

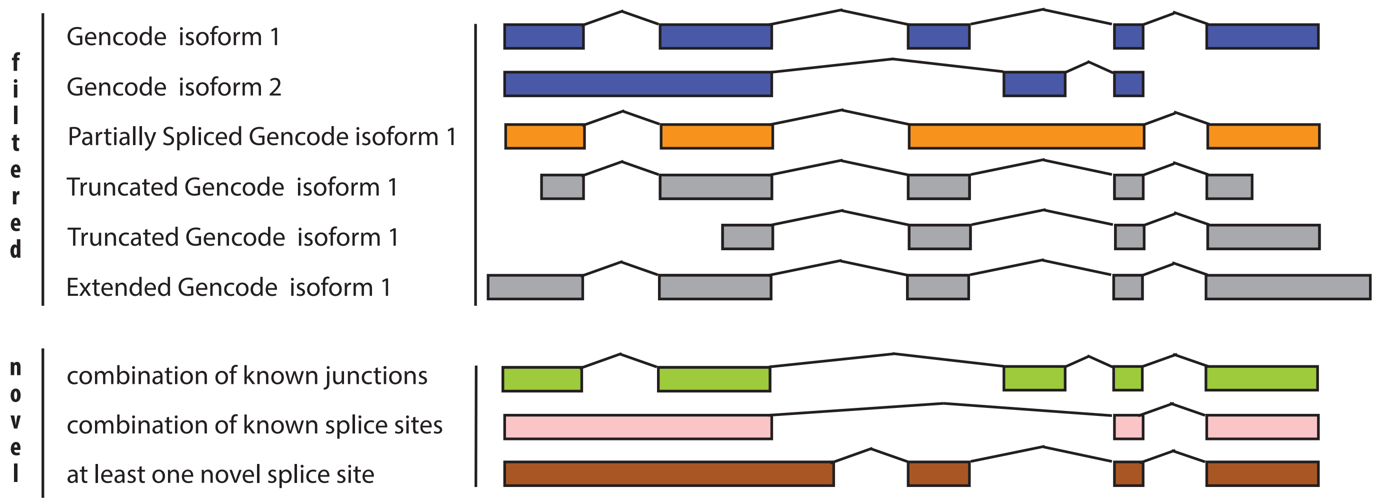

b

	Total	Splice junctions in Illumina data	CAGE peak	Polyadenylation site	Final	Final % of total
known gencode	33,002	33,002	20,533	14,908	11,186	34%
novel - combination of known junctions	3,063	3,068	2,644	1,939	1,696	55%
novel - combination of known splice sites	2,111	1,905	1,906	1,366	1,115	53%
novel - at least one novel splice site	5,507	3,166	4,614	3,553	1,577	29%
novel - total	10,681	8,134	9,164	6,858	4,388	41%

c

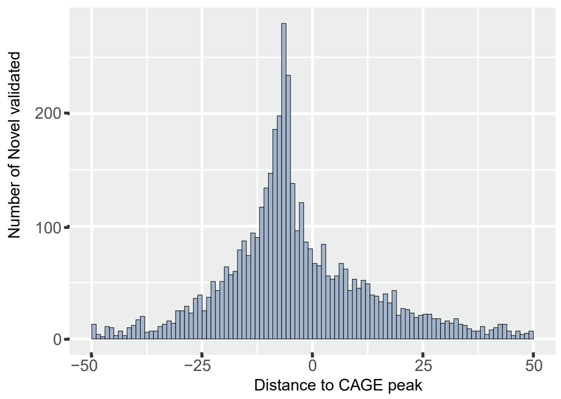

d

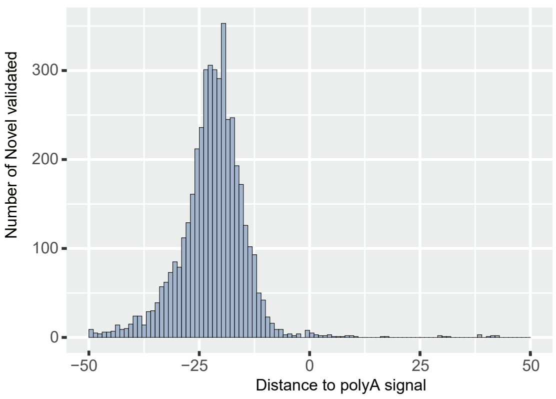

e

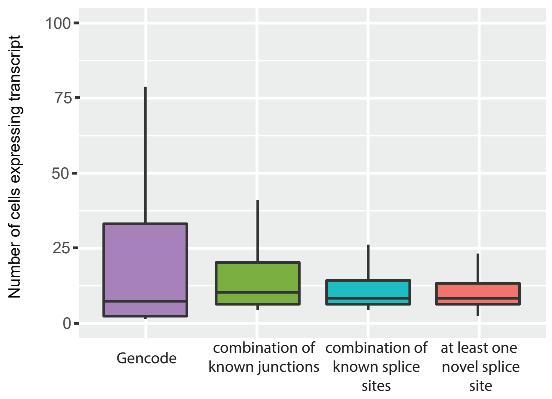

Supplementary Figure 6. Identification of potential novel transcript isoforms.

(a) Definition of novel transcript isoforms. Before we analyzed our dataset for potentially novel transcript isoforms, we removed transcripts that contain only splice junctions that are found in an annotated Gencode isoform. This filter removes full-length, truncated, extended and partially spliced Gencode transcripts. Three different categories of novel transcript isoforms were considered: **"Combination of known junctions"** refers to transcripts in which all splice junctions are annotated in at least one of the Gencode isoforms, but all junctions are not found in a single gencode transcript; **"Combination of known splice sites"** refers to transcripts in which at least one non-Gencode splice junction is found. The novel splice junctions must involve splice sites that are found in the annotated Gencode isoforms. **"At least one novel splice site"** refers to transcripts with at least one splice site that is not found in Gencode. **(b)** Table shows the number of isoforms that pass the following filters: (i) identification of all splice junctions either in gencode transcripts or in a mouse E18 mouse brain short read Illumina dataset; (ii) presence of a CAGE peak within 50 nt. of the 5' of the cDNA (iii) presence of a polyadenylation site within 50 nt. of the 3' end of the cDNA (see methods section for details). (**c,d**) Distance of 5' and 3' ends of 4,388 novel transcript isoforms from known CAGE peaks (c) or polyadenylation sites (d). **(e)** Boxplot showing the distribution of the number of cells that express identified Gencodes transcripts (33,002) and transcripts from the different categories of filtered novel isoforms (only known exons-exon junctions, n=1,696; only known splice sites, n=1,115; at least one novel splice site, n=1,577). All novel isoforms are expressed in at least two cells. Boxes represent the 25% quantile to 75% quantile range, upper and lower edges of notches are median +/- 1.58 * IQR / sqrt(n) (IQR: inter quantile range, n: sample size).

Supplementary Figure 7

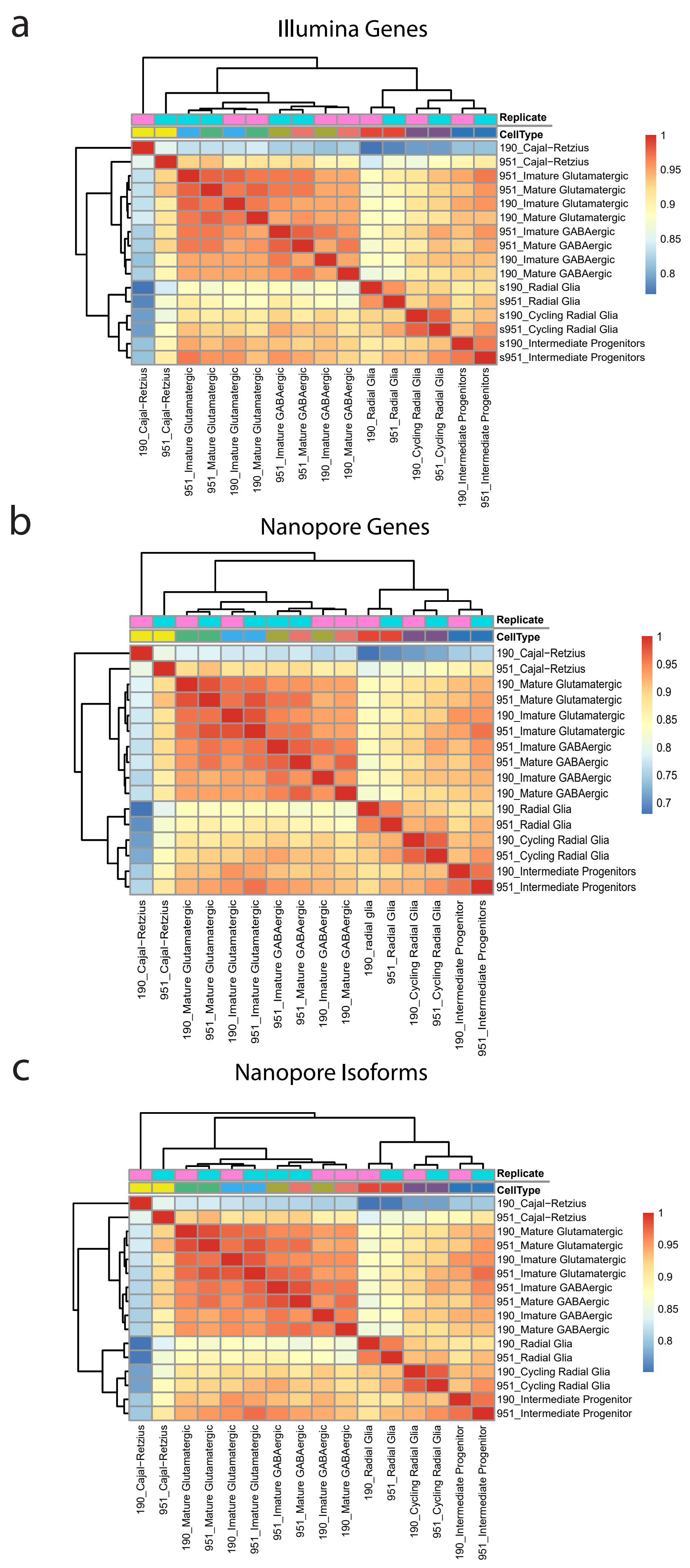

Supplementary Figure 7. Correlation between 190 cell and 951 cell replicates.

Correlations of gene expression based on Illumina short read (**a**), Nanopore long read (**b**) or Nanopore long read transcript isoform (**c**) for the expression measured between the 190 cell and the 951 cell datasets are shown for the different cell types (Fig. 2d). Correlations shown in the heatmaps are Pearson correlation coefficients.

Supplementary Figure 8

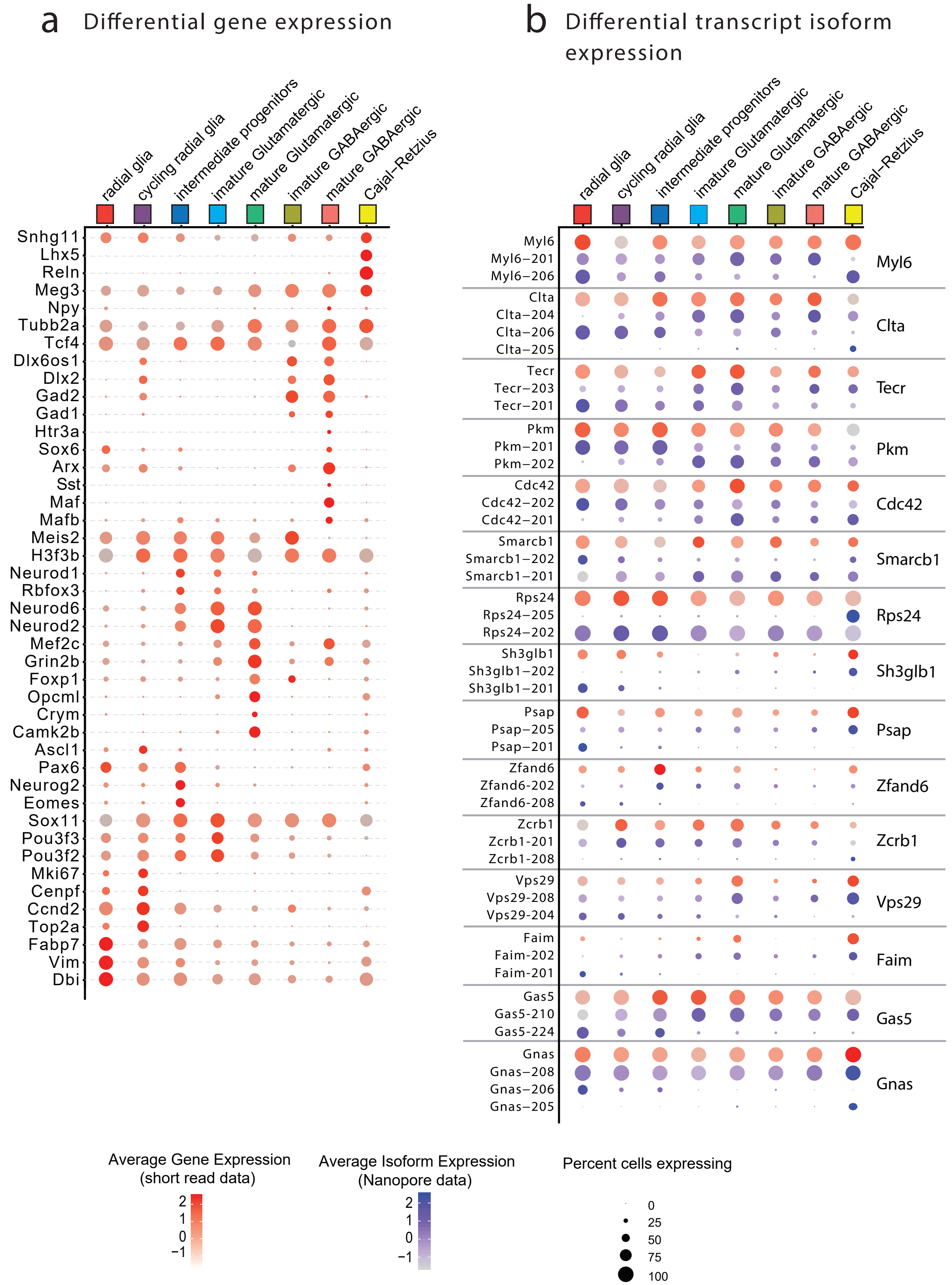

Supplementary Figure 8. Differential gene and transcript isoform expression in subtypes of neurons.

(a, b) Expression of selected genes (a) and isoforms (b) in 8 different cell types (Fig. 2d). **(a)** Illumina short read sequencing gene expression for a subset of the genes markers differentially expressed between clusters. **(b)** Gene (red dots) and isoform (blue dots) expression for selected genes that show differential expression of isoforms between neuronal subtypes (clusters). Dot size relates to the percentage of expressing cells in each cluster while color intensity relates to the expression level. Transcript names are from Ensembl. The full list of differentially expressed genes and isoforms is in Supplementary Table 1.

Supplementary Figure 9

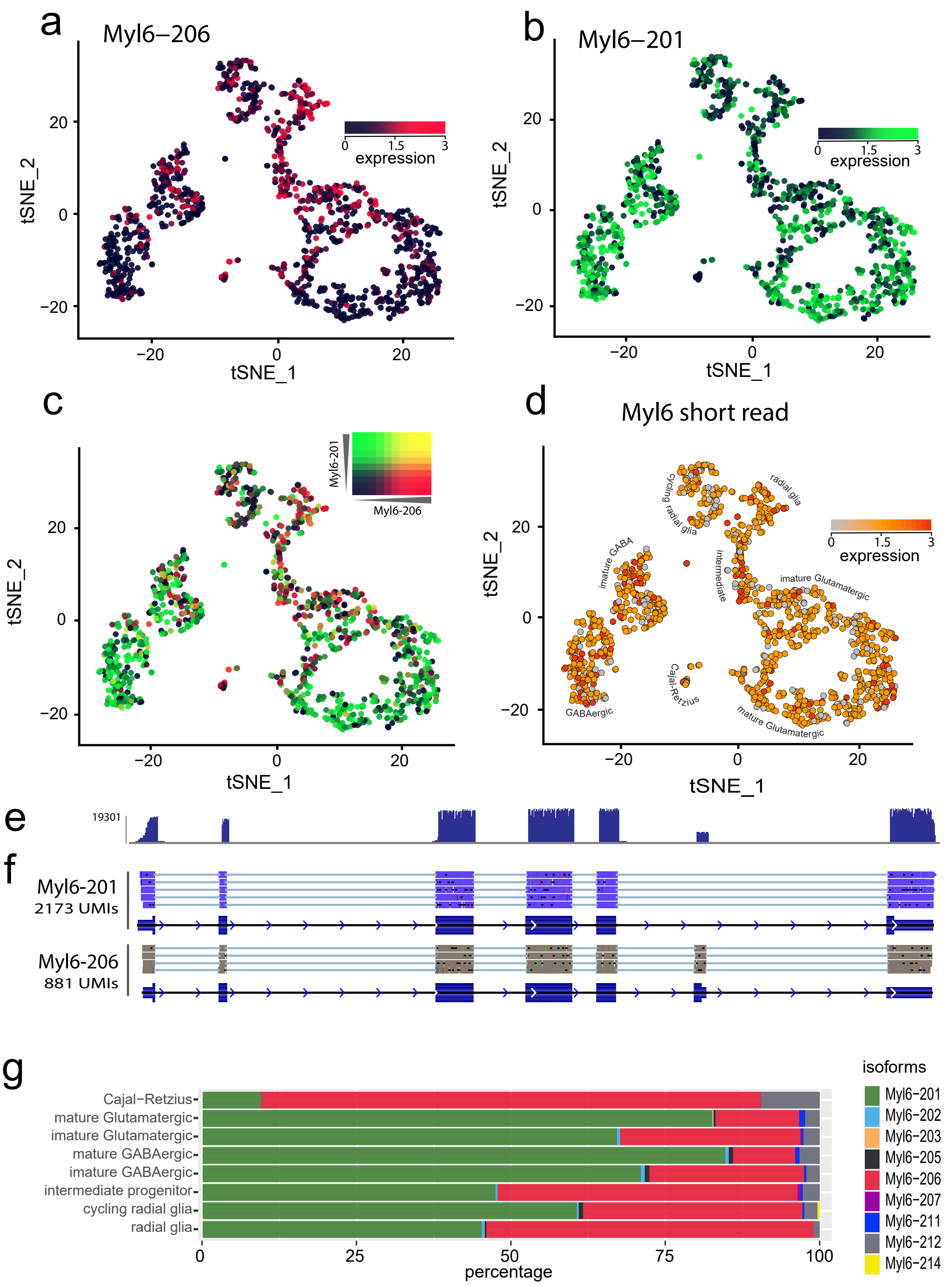

Supplementary Figure 9. Myl6 alternate splice variants are differentially expressed during neuronal maturation.

(a-c) Isoform switch of the essential (alkaline) Myosin light chain 6 during neuronal maturation visualized on the t-SNE plot of Fig. 1e (1,121 cells). (**a**) Myl6-206 (ENSMUST00000218127.1), (**b**) Myl6-201 (ENSMUST00000164181.1), (**c**) Overlay Myl6-201/Myl6-206. **(d)** Myl6 gene expression from Illumina short read data. **(e)** Nanopore read density distribution over the Myl6 gene. **(f)** Genome aligned Myl6 reads for two UMIs. The number of UMIs for the integrated 1,121 cell dataset is indicated for each isoform. (e) and (f) are screenshots of the aligned Nanopore Bam files visualized with IGV (Integrated Genome Viewer, UCSC Santa Cruz). **(g)** Relative expression of known Ensembl Myl6 transcript isoforms in different cell types of the brain (clusters of Fig. 2d).

SUPPLEMENTARY NOTES

Supplementary Note: Identification of novel transcript isoforms.

Single cell long read sequencing is a powerful approach to detect cell type selective expression of transcript isoforms. However, the definition of any novel transcript isoform requires a careful consideration of potential artifacts that are inherent to all RNA-seq library preparations that involve RT and PCR amplification steps. While most PCR artifacts can be eliminated when UMIs are used, identification of RT artifacts is, in general, more difficult. Reverse transcriptases tend to switch templates between homologous sequences[1] resulting in deletions (intramolecular) or chimeric cDNA (intermolecular).

When RNA-seq libraries are prepared from purified RNA, the sample is denatured to remove RNA secondary structure, snap cooled, and RT is carried out at ≥ 37°C. In scRNA-seq with the most widely used systems (e.g. 10x Genomics Chromium), cells are co-encapsulated with RT reagents and RT-primer beads into emulsion droplets where cells are lysed. Thus, reverse transcription is initiated at room temperature without disrupting the RNA secondary structure and proceeds at low stringency until the emulsion is recovered and heated to complete the RT. Inter and intramolecular secondary structure of RNA is known to favor RTase template switching [1]. The low stringency initiation of the RT within the droplets also leads to false priming of the oligo-dT primers on internal A-rich sequences. We indeed observed such RT priming on A-rich sequences mainly within 3' UTRs or intronic sequences of partially spliced mRNAs. Thus, single cell cDNA has typically a higher fraction of artifactual cDNA than cDNA prepared from purified mRNA under well controlled conditions.

To limit this issue, we required at least five UMIs per isoform and filtered our dataset of potential novel transcript isoforms extensively (Supplementary Fig. 6a): (i) We eliminated transcripts that contain only splice junctions that are found in an annotated Gencode transcript. This step eliminates Gencode

transcripts with lacking, truncated or extended terminal exons as well as partially spliced Gencode transcripts; (ii) When all splice junctions of a potentially novel transcript were included in another novel transcript that has more exons, the shorter form was discarded; (iii) We removed mono-exonic transcripts. Three steps of validation were then applied to the remaining 10,681 potential novel isoforms (Supplementary Fig. 6, Supplementary Table 2): novel splice junctions had to be found in a mouse brain short read dataset, 5' ends had to be close (+/- 50 nt.) to an existing CAGE tag and a 3' polyadenylation site had to be present (+/- 50 nt.) (Supplementary Fig. 6). 41% (4,388 out of 10,681) of the novel isoforms passed all those validation steps. The fact that only 34% (11,186 out of 33,002) of know Gencode transcripts pass, suggests that we likely also eliminated valid novel transcripts with those filters (Supplementary Fig. 6). The 4,388 novel isoforms that passed all our validation filters suggest that a huge diversity of transcripts isoforms remains to be discovered and that long read single cell sequencing will prove a valuable tool to define the diversity of known and novel transcript isoforms in single cells.

However, we cannot exclude that some artefactual sequences passed our filters (e.g. RT template switching also present in Illumina data), despite the use of UMIs to reduce PCR artefacts. At this point, it is wise to validate each novel transcript isoform discovered in a single-cell sequencing study extensively before a biological role of such a novel isoform is implied.

Supplementary References

1. Cocquet, J., Chong, A., Zhang, G. & Veitia, R.A. *Genomics* **88**, 127-131 (2006).
