# Supplementary material for: High throughput error corrected Nanopore single cell transcriptome sequencing
Source: Nat Commun. 2020 Aug 12;11:4025. doi: 10.1038/s41467-020-17800-6 (PMC7423900; doi:10.1038/s41467-020-17800-6)
Supplement: Supplementary file 5 — Reporting Summary [file 41467_2020_17800_MOESM5_ESM.pdf]

nature research

Corresponding author(s): Pascal Barbry

Last updated by author(s): Jul 14, 2020

Reporting Summary

Nature Research wishes to improve the reproducibility of the work that we publish. This form provides structure for consistency and transparency in reporting. For further information on Nature Research policies, see our Editorial Policies and the Editorial Policy Checklist.

Statistics

For all statistical analyses, confirm that the following items are present in the figure legend, table legend, main text, or Methods section.

n/a	Confirmed	
☐	☒	The exact sample size (*n*) for each experimental group/condition, given as a discrete number and unit of measurement
☐	☒	A statement on whether measurements were taken from distinct samples or whether the same sample was measured repeatedly
☐	☒	The statistical test(s) used AND whether they are one- or two-sided<br>*Only common tests should be described solely by name; describe more complex techniques in the Methods section.*
☒	☐	A description of all covariates tested
☐	☒	A description of any assumptions or corrections, such as tests of normality and adjustment for multiple comparisons
☐	☒	A full description of the statistical parameters including central tendency (e.g. means) or other basic estimates (e.g. regression coefficient) AND variation (e.g. standard deviation) or associated estimates of uncertainty (e.g. confidence intervals)
☐	☒	For null hypothesis testing, the test statistic (e.g. *F*, *t*, *r*) with confidence intervals, effect sizes, degrees of freedom and *P* value noted<br>*Give P values as exact values whenever suitable.*
☒	☐	For Bayesian analysis, information on the choice of priors and Markov chain Monte Carlo settings
☒	☐	For hierarchical and complex designs, identification of the appropriate level for tests and full reporting of outcomes
☒	☐	Estimates of effect sizes (e.g. Cohen's *d*, Pearson's *r*), indicating how they were calculated

Our web collection on statistics for biologists contains articles on many of the points above.

Software and code

Policy information about availability of computer code

Data collection	Data were processed with R codes, largely based on published Bioconductor packages.
Data analysis	Custom R and JAVA code that were used have been deposited on Github (https://github.com/ucagenomix/sicelore). The version of programs/ packages were: minimap2 v2.17, Cell Ranger 2.0.0, R/Bioconductor (version 3.5.2), Seurat R package (version 3.1.4), sicelore 1.0.

For manuscripts utilizing custom algorithms or software that are central to the research but not yet described in published literature, software must be made available to editors and reviewers. We strongly encourage code deposition in a community repository (e.g. GitHub). See the Nature Research guidelines for submitting code & software for further information.

Data

Policy information about availability of data

All manuscripts must include a data availability statement. This statement should provide the following information, where applicable:

- Accession codes, unique identifiers, or web links for publicly available datasets
- A list of figures that have associated raw data
- A description of any restrictions on data availability

Single-cell data that support the findings of this study have been deposited in GEO with the accession code GSE130708 (https://www.ncbi.nlm.nih.gov/geo/query/acc.cgi?acc=GSE130708). The data are now public.

Field-specific reporting

Please select the one below that is the best fit for your research. If you are not sure, read the appropriate sections before making your selection.

[x] Life sciences   [ ] Behavioural & social sciences   [ ] Ecological, evolutionary & environmental sciences

For a reference copy of the document with all sections, see nature.com/documents/nr-reporting-summary-flat.pdf

Life sciences study design

All studies must disclose on these points even when the disclosure is negative.

Sample size	The consistence of cell-type identification was compared with already published materials. We followed the rationale previously used by Gupta et al (https://www.nature.com/articles/nbt.4259) who also described their method from a unique biological sample. In our case, it provided two independent batches of cells that contained 190 and 951 cells.
Data exclusions	No data was excluded from the analysis
Replication	Distinct collections of cells from the same tissue were independently analyzed. All attempts at replication were successful.
Randomization	Randomization was not relevant for this study, which describes the development of a new method. No treatment or drug was applied on our biological samples
Blinding	Blinding was not possible because the wet lab experiments were done by one single person.

Reporting for specific materials, systems and methods

We require information from authors about some types of materials, experimental systems and methods used in many studies. Here, indicate whether each material, system or method listed is relevant to your study. If you are not sure if a list item applies to your research, read the appropriate section before selecting a response.

Materials & experimental systems

n/a	Involved in the study	
[x]	[ ]	Antibodies
[x]	[ ]	Eukaryotic cell lines
[x]	[ ]	Palaeontology and archaeology
[x]	[ ]	Animals and other organisms
[x]	[ ]	Human research participants
[x]	[ ]	Clinical data
[x]	[ ]	Dual use research of concern

Methods

n/a	Involved in the study	
[x]	[ ]	ChIP-seq
[x]	[ ]	Flow cytometry
[x]	[ ]	MRI-based neuroimaging
