# Supplementary material for: High throughput error corrected Nanopore single cell transcriptome sequencing
Source: Nat Commun. 2020 Aug 12;11:4025. doi: 10.1038/s41467-020-17800-6 (PMC7423900; doi:10.1038/s41467-020-17800-6)
Supplement: Supplementary file 6 — Description of Additional Supplementary Files [file 41467_2020_17800_MOESM6_ESM.pdf]

Title: Supplementary Data 1.

Description: Table of differentially expressed features in the clusters shown in Fig. 2d; sheet 1: Illumina short read data; sheet 2: Nanopore long read data, each cluster was compared with rest of cells; sheet 3: Nanopore long read data, pairwise comparison of clusters. Analysis was performed using the default parameters from Seurat::FindMarkers, using a Wilcoxon Rank Sum test. p-value adjustment was performed using Bonferroni correction based on the total number of genes in the dataset.

Title: Supplementary Data 2.

Description: Table with identified Gencode vM18 annotated transcript isoforms and potential novel isoforms.

Title: Supplementary Data 3.

Description: E18.mouse.brain.isoforms.gff.zip, GFF file of identified Gencode vM18 annotated transcripts isoforms and the 4,388 filtered potential novel transcript isoforms.
